# Biomarkers of exposure and potential harm in exclusive users of electronic cigarettes and current, former, and never smokers

**DOI:** 10.1007/s11739-023-03294-9

**Published:** 2023-05-30

**Authors:** Linsey E. Haswell, Nathan Gale, Elaine Brown, David Azzopardi, Michael McEwan, Jesse Thissen, Filimon Meichanetzidis, George Hardie

**Affiliations:** BAT (Investments) Limited, Regents Park Road, Millbrook, Southampton, SO15 8TL UK

**Keywords:** Biomarkers of exposure, Biomarkers of potential harm, Electronic cigarettes, Tobacco harm reduction

## Abstract

Electronic cigarette (EC) aerosol emissions generally contain fewer and lower concentrations of harmful and potentially harmful constituents, compared with cigarette smoke. Further studies are needed to establish whether decreased emissions translate to reduced health risks for EC users. In a cross-sectional study, biomarkers of exposure (BoE) to certain tobacco smoke toxicants and biomarkers of potential harm (BoPH), associated with biological processes linked to the potential development of smoking-related diseases and oxidative stress, were assessed in solus Vuse ECs users and current, former, and never smokers. In total, 213 participants were enrolled, and smoking status was confirmed by urinary cotinine, exhaled carbon monoxide, and N-(2-cyanoethyl)valine levels (EC users and former smokers only). During confinement participants used their usual product (EC or cigarette) as normal and BoE and BoPHs were assessed via blood, 24-h urine, and physiological assessment. Significantly lower levels of all urinary BoE; MHBMA, HMPMA, 3-HPMA, NNN, 3-OH-B[a]P, S-PMA, NNAL (all *p* < 0.0001), and TNeq (*p* = 0.0074) were observed in EC users when compared with smokers. Moreover, significantly lower levels were observed in EC users for 3 of the 7 BoPH measured, carboxyhaemoglobin (*p* < 0.0001), soluble intercellular adhesion molecule-1 (*p* = 0.0028), and 11-dehydrothromboxane B2 (*p* = 0.0012), when compared with smokers. As compared with smokers, solus Vuse EC users have significantly lower exposure to tobacco toxicants for the BoE, and 3 BoPH measured. These results add to the weight of evidence supporting EC as part of a tobacco harm reduction strategy.

**Supplementary Information:**

The online version contains supplementary material available at 10.1007/s11739-023-03294-9.

## Introduction

The health risks associated with smoking include cardiovascular disease, chronic obstructive pulmonary disease, and lung cancer [1]. These risks are primarily due to the long-term inhalation of cigarette smoke, which contains more than 6500 identified chemicals [2] and multiple harmful and potentially harmful constituents (HPHCs) [3] created and released during the combustion of tobacco [1, 4–8]. Quitting smoking greatly reduces the relative risks of smoking-related diseases [1], which has led to the public health priority of reducing the health burden of cigarette smoking by encouraging smoking abstinence [9]. Despite these efforts, smoking rates in adult populations worldwide remain around 20%, although prevalence is declining in many countries [10]. Key factors in the development of smoking-related diseases have been identified as oxidative stress, DNA damage, and inflammation [5, 11, 12], but the finer mechanisms are not yet fully elucidated [5].

Electronic cigarettes (EC) typically provide a nicotine-containing aerosol with significantly fewer and generally substantially lower levels of HPHCs than cigarette smoke [13–18]. Many health bodies now support the use of ECs as an alternative to smoking conventional cigarettes [19–22] as part of a tobacco harm reduction (THR) strategy, first proposed by the US Institute of Medicine in 2001 [5]. The validity of this approach, which aims to help those seeking an alternative to smoking to choose instead a less harmful alternative product [5], is supported by the findings of cross-sectional and longitudinal studies [5, 23–26].

Although the reduced numbers and concentrations of HPHCs in EC aerosol relative to cigarette smoke have been well established [13–18], it is not yet clear whether these reductions may translate into measurable changes in the health risks of EC use as compared to cigarette smoking. Recent studies have begun to assess various biomarkers of exposure (BoE) to determine the internal dose of HPHCs received by the product user. Short-term confinement studies have shown marked reductions in BoE among people who smoke switching to exclusive EC use [27–31]; similarly, cross-sectional studies [32, 33] have reported substantially lower concentrations of BoE among people who use EC than among people who smoke. By contrast, fewer studies have assessed differences in biomarkers of potential harm (BoPH) [32] which give an indication of mechanistic and physiological effects caused by exposure, including changes in biological pathways and functions, and/or clinical symptoms associated with harm [34]. Studies of BoPH can help to establish whether novel tobacco and nicotine products offer reduced health risks relative to smoking [35] and can inform regulatory processes [5, 36].

The aim of this study was to assess selected BoPH, BoE, and physiological measures in individuals who had been exclusively using commercially available EC (Vuse, Nicoventures Trading Ltd, London, UK) for at least 6 months, individuals who currently smoke, former, and never smokers.

## Methods

### Study design

This cross-sectional confinement study was conducted among EC users and current, former, and never smokers attending a single study centre in London, UK, between March and September 2021. The Research Ethics Committee of the NHS Health Research Authority, South Central, Berkshire B, UK gave favourable opinion (equivalent to Institutional Review Board approval) for the study (reference number 21/SC/0005), and all participants provided written informed consent before undergoing any procedures, including screening assessments. The study design and protocol has been described in full elsewhere [37]. The study was conducted in accordance with the Declaration of Helsinki and Good Clinical Practice, and is reported in accordance with the International Council on Harmonisation guidelines. The trial has been registered with the International Standard Registered Clinical/Social Study Number registry (ISRCTN58921739).

### Study objectives

The primary objective was to quantitatively assess differences between EC users and current smokers in total 4-(methylnitrosamino)-1-(3-pyridyl)-1-butanol (NNAL), fractional exhaled nitric oxide (FeNO), 8-epi-prostaglandin F_2α_ Type III (8-epi-PGF_2α_ Type III), carboxyhaemoglobin (COHb), white blood cell count (WBC), soluble intercellular adhesion molecule-1 (sICAM-1), and high-density lipoprotein (HDL). The secondary objective was to quantitatively assess differences between EC users and current smokers in total nicotine equivalents (nicotine, cotinine, 3-hydroxycotinine, and their glucuronide conjugates) [TNeq], monohydroxybutenylmercapturic acid (MHBMA), 3-hydroxy-1-methylpropylmercapuric acid (HMPMA), 3-hydroxypropylmercapturic acid (3-HPMA), total N-nitrosonornicotine (NNN), 3-hydroxybenzo[*a*]pyrene (3-OH-B[*a*]P), and S-phenylmercapturic acid (S-PMA), 11-dehydrothromboxane B2 (11-dTX B2), forced expiratory volume in 1 s as % of predicted (FEV1%pred) and carotid intima-media thickness (CIMT), as well a quality-of-life questionnaire. Differences in all study endpoints between EC users and former smokers, and between EC users or former smokers and never smokers were qualitatively assessed.

### Study participants

Potential participants were identified by Richmond Pharmacology Limited (London, UK) through advertising campaigns, database contact, referral scheme, and social media channels. An external recruitment agency was used to identify potential EC user participants. Eligible participants were healthy adults (age 19–55 years) exclusive users of Vuse ECs, smokers (≥ 10 cigarettes/day), former smokers, and never smokers. The lower age limit of 19 was selected to ensure participants who smoke had been legally smoking for at least 1 year prior to screening, as the minimum legal age required to purchase or use tobacco products in the UK is 18 years old. The upper age restriction was 55 years to limit the influence of age-related confounding effects on BOPH. Exclusive EC use was defined as self-reported daily use of Vuse EC devices for a minimum of 6 months prior to screening and was confirmed by measurements of urinary cotinine > 200 ng/mL and exhaled breath carbon monoxide (eCO) < 7 ppm [38]. Current smoking status was defined as self-reporting smoking of at least ten cigarettes per day for a minimum of 1 year prior to screening and was confirmed by urinary cotinine > 200 ng/mL and eCO ≥ 7 ppm. Former smokers self-reported having quit smoking for at least 6 months and current non-smoking status was confirmed by urinary cotinine < 200 ng/mL and eCO < 7 ppm. Never smokers self-reported smoking fewer than 100 cigarettes in their lifetime and none within the 6 months before screening, along with urinary cotinine < 200 ng/mL and eCO < 7 ppm. Compliance with smoking abstinence in the previous 6 months among EC users and former smokers was assessed by measurement of *N*-(2-cyanoethyl)valine (CEVal) [39].

The full inclusion and exclusion criteria have been described previously [37]. The main inclusion criteria were general good health and no clinically relevant abnormal findings on physical examination, vital signs assessment, electrocardiogram, clinical laboratory evaluations, lung function tests, or medical history. The main exclusion criteria were females who were pregnant/breastfeeding; blood donation (≥ 400 mL) in the 90 days before screening; and acute illness requiring treatment in the 28 days before screening. In addition, participants were asked to avoid alcohol completely for a period of at least 24 h; eating food containing poppy seeds for 3 days as this can lead to a positive opiate result in the drugs of abuse test; and eating or cooking cruciferous vegetables which is associated with induction of cytochrome P450 1A2 and grilled, fried or barbequed food which could influence the assessment of BOE, for 48 h prior to attending the clinic.

### Study protocol

On study day 1, potential participants were invited to attend the clinic for screening, which included physical and vital signs examinations, routine clinical laboratory testing, alcohol and drug consumption testing, and pregnancy testing. Nicotine use and smoking status were confirmed by eCO and cotinine tests. Extent of tobacco and nicotine use was assessed via a questionnaire (Supplementary Information). Individuals who met the inclusion criteria were enrolled after screening and immediately began the study.

Participants supplied their own ECs or cigarettes, sufficient to cover their typical usage for the 24-h study period, and were asked to use them as and when they normally would. During this time, 24-h urine samples were collected, and blood sampling (for BoE, BoPH, and CEVal), physiological assessments, and a quality-of-life questionnaire (RAND 36-Item Short Form Health Survey questionnaire) [40] were conducted. After completion of health checks and safety assessments at the end of day 2, participants were discharged from the clinic. A follow-up 7 days after discharge was performed by telephone call to collect information on the status of any ongoing adverse events (AEs) at discharge and any new AEs experienced post-discharge.

Participants were allowed to withdraw from the study at any time and for any reason. They could also be withdrawn from the study by the principal investigator, for example, for health reasons or protocol deviations.

### Study products

No study products were provided; instead, participants supplied their own ECs and e-liquid cartridges and cigarettes for use during the study. The recruited smokers used their own brand of cigarette. The recruited EC users were self-reported exclusive users of commercially available Vuse ePod or Vuse ePen3 ECs (Nicoventures Trading Ltd, UK). Both ECs comprise a reusable device containing a 350-mAh (ePod) or 650-mAH (ePen3) rechargeable battery and a disposable cartridge containing e-liquid (1.9 or 2.0 ml, respectively). The two ECs were used only with the manufacturer’s recommended cartridges. The design, materials, and aerosolization performance of the Vuse ePod are substantially similar to the Vuse Alto (R. J. Reynolds Vapor Company.). Vuse ePod cartridges contain a ceramic wick, and a flat metallic heating element. Vuse ePen3 cartridges contain a silica a rope wick and a NiCr coil. The recruited EC used their own Vuse e-liquids. Vype was rebranded to Vuse in the UK on 1st Apr 2021, during the clinical conduct phase of the study.

### Compliance measurements

For EC users and former smokers, smoking abstinence in the previous 6 months was confirmed by measurement of a haemoglobin adduct of acrylonitrile (CEVal) in erythrocytes as described [41]. Acrylonitrile is found in tobacco smoke, but not in aerosol from ECs [16] and tobacco smoke is the major non-occupational source for acrylonitrile exposure. The CEVal-compliant population was defined as a subset of the per-protocol population, and included only those Vuse users and former smokers whose blood CEVal level was below 54 pmol/g Hb. The threshold of CEVal used to determine compliance has been described previously [39, 42].

### Biomarkers of exposure

BoE were selected from the WHO Study Group on Tobacco Product Regulation (Tob Reg) initial list of priority toxicants [43]. For two of the listed toxicants, acetaldehyde and formaldehyde, there are no reliable BoE at present; therefore, levels of crotonaldehyde were assessed via HMPMA instead. The BoE assessed were HMPMA, 3-HPMA, 3-OH-B[*a*]P, MHBMA, total NNN, S-PMA, TNeq, and total NNAL. The analysis was conducted at Analytisch-biologisches Forschungslabor (ABF) GmbH, Planegg, Germany as previously described [37, 44, 45].

### Biomarkers of potential harm

The BoPH selected for this study were HDL, sICAM-1, COHb, 11-dTX B2, WBC and CIMT, FeNO and FEV_1_%pred, and 8-epi-PGF_2α_ Type III. NNAL is a BoE for the tobacco-specific nitrosamine 4-(methylnitrosamino)-1-(3-pyridyl)-1-butanone (NNK) and an animal carcinogen [6]. The biological processes associated with each BOPH have been described previously [37]. Urinary NNAL is associated with lung cancer risk [46], and, therefore, is considered a BoPH for lung cancer [47, 48].

The BoPH analytical methods have been described in detail previously [37]. In brief, the urinary eicosanoids 11-dTX B2 and 8-epi-PGF_2α_ type III and COHb were measured at ABF. Celerion AG (Zurich, Switzerland) conducted serum sICAM-1 and urinary creatinine analyses. WBC and HDL were measured in blood and serum, respectively, by The Doctors Laboratory (London, UK). CIMT was measured by ultrasound. The assessment was performed on a 10 mm section of the distal portion of the common carotid artery, on both sides of the neck, at least 5 mm from the carotid bulb. FEV_1_ was measured by spirometry (without a bronchodilator) in accordance with procedures of the American Thoracic Society/European Respiratory Society [49], and FEV_1_%pred values were standardised to the Global Lungs Initiative predictive values. Participants were not allowed to eat for 2 h, or smoke or vape for 1 h, prior to spirometry assessments. Levels of nitric oxide in exhaled breath were determined by assessing FeNO using the Vivatmo Pro (Bosch Healthcare Solutions, Waiblingen, Germany).

### Quality-of-life questionnaire

Self-reported quality of life was assessed via the RAND 36-Item Short Form (SF-36) Health Survey questionnaire [40].

### Safety

Participant safety was monitored in clinic by vital signs assessment, physical examination, and clinical laboratory assessments, and by telephone follow-up 7 days after discharge. All AEs were recorded and coded in accordance with the Medical Dictionary for Regulatory Activities version 24.0, with severity classified as mild, moderate, or severe.

### Statistical analysis

To determine sample size, all primary endpoints were assessed by literature review. Of these, sICAM-1 showed the most variability in terms of mean ratios and coefficients of variation (CVs). The ratio of means for sICAM-1 between current and former smokers was 0.697‒0.847 in identified studies, and CVs were 24.5‒34.1%. Based on these data, a sample size calculation was performed using PROC POWER (SAS version 9.4) to enable assessment of differences between EC users and current smokers in this study. It was assumed that EC users have a ratio below 1 when compared to smokers and set a mean ratio of 0.847 with CV of 27.1‒32.8% based on data from Haswell et al. [50]. These values would yield *β* = 0.2 and *α* = 0.05. Thus, it was determined the study would require 84‒120 participants to complete across the two groups to demonstrate a significant difference. This number would provide power of 0.806. Since the split between EC users and current smokers was not planned to be equal, the total for these combined groups was set at a minimum of 120.

Data for all urinary biomarkers were converted to values per 24-h period, by multiplying the reported concentration by the volume of urine collected from the subject in the 24-h period. Primary and secondary endpoints were summarised using descriptive statistics (n, mean and standard deviation) and presented by study arm.

Statistical analysis was performed on the means of Vuse user and smoker groups using an Analysis of Covariance (ANCOVA) general linear model. The ANCOVA model was fitted with the endpoint result as the dependent variable, Group and Sex were included as fixed effects and Age was included as a covariate. Multiple comparisons were performed for the primary objective assessments as multiple endpoints were tested using least-squares mean (LS-mean). To control the type-I error rate, the Bonferroni correction method was applied. The threshold of statistical significance was divided by the number of endpoints tested, i.e., 0.05/7. If the p value was below 0.00714, the null hypothesis was rejected, and the means in Vuse user and smoker groups were considered statistically different. For secondary endpoints, no adjustment was made for multiplicity, and thus, the threshold of statistical significance was equal to 0.05.

Descriptive summaries and data analysis were performed on the per-protocol and the CEVal-compliant populations. Analyses were completed using SAS version 9.4 software.

## Results

### Demographics of the study participants

The first participants were enrolled on 26 March 2021 and the last follow-up call was performed on 09 September 2021. The study recruited 213 participants. Participants were not randomised but were enrolled directly into each study group, Supplementary Fig. 1 shows the deposition of the participants into the study arms and populations. The safety population comprised of 99 Vuse users, 40 smokers, 37 former smokers, and 37 never smokers. Of the 213 participants, 194 completed the study. One participant was withdrawn from the per-protocol and CEVal-compliant populations of the Vuse users group due to a major protocol deviation (failed Exclusion Criterion: Participants who have used any form of tobacco or nicotine-containing product, other than the Vuse e-Pen3 and/or e-Pod, within the 6 months prior to screening) and the remaining 18 participants were lost to follow-up. Participants who completed the in-clinic part of the study (with no major protocol deviations) were included in the per-protocol population even if they were lost to follow up.

Basic participant demographic details for the CEVal-complaint population are presented in Supplementary Table 1 and the per-protocol population in Supplementary Table 2. In the CEVal-compliant population, Vuse users, smokers, and never smokers were similar in age (mean ± SD: 29.6 ± 8.26, 29.5 ± 6.57, and 30.4 ± 7.64 years, respectively), the oldest group of participants were former smokers (mean ± SD, 35.8 ± 9.73). The overall proportion of females in the CEVal-compliant population was 41.8% and the proportion in the smoker and never smoker groups were relatively similar. The proportion of females in the CEVal-compliant Vuse users’ group was slightly lower at 35.5% and higher in the former smoker group at 51.4%. There were no notable differences in body mass index between study groups.

### Compliance

Compliance with self-reported solus use of e-cigarettes and smoking abstinence was assessed by CEVal, a haemoglobin adduct of acrylonitrile, in the Vuse user and former smoker groups. Five participants in the Vuse user and two participants in the former smoker groups were determined to have CEVal levels above the threshold and were excluded from the CEVal-compliant population.

### Biomarkers of exposure

**Fig. 1:**
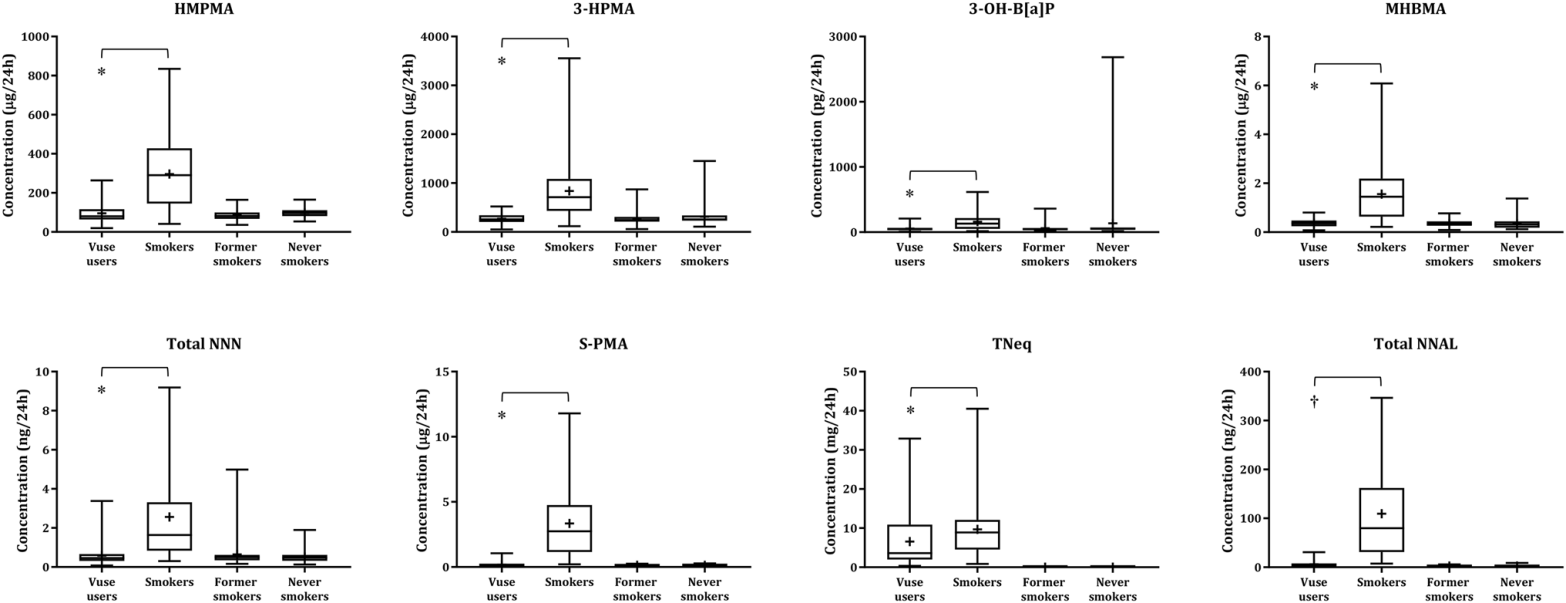
Biomarkers of exposure boxplots by study groups for the CEVal-compliant population. The bar inside the box is the median and the arithmetic mean is the cross inside the box. The upper (75th percentile) and lower (25th percentile) sides of the box represent the interquartile range; the lower whisker is the minimum and the upper whisker is the maximum. *Secondary endpoint with threshold of statistical significance = 0.05. †Primary endpoint with threshold of statistical significance = 0.00714, to account for multiple endpoint testing

The levels of the eight urinary tobacco toxicant BoE assessed in the CEVal-compliant participants are shown in Fig. 1. The statistical analyses of the BoE were performed on the Vuse user and smoker groups, as per the SAP, and the descriptive statistics and statistical analyses of the differences between these groups in the per-protocol and CEVal-compliant populations are presented in Table 1. Significantly lower levels of all the BoE; HMPMA, 3-HPMA, 3-OH-B[a]P, MHBMA, total NNN, S-PMA, total NNAL (all *p* < 0.0001), and TNeq (*p* = 0.0074) were observed in the CEVal-compliant Vuse users group when compared with people who smoke. The per-protocol population BoE levels are presented in Supplementary Fig. 2. The BoE descriptive statistics of the former smoker and never smoker groups (both per-protocol and CEVal-compliant) are presented in Supplementary Table 3.

**Table 1:** Biomarkers of exposure descriptive statistics and statistical analysis of the per-protocol and CEVal-compliant populations

Biomarker of exposure (Units)	Associated		Per-protocol population				CEVal-compliant population			
	Toxicant	Group	*N*	Mean	SD	*p* Value*	*N*	Mean	SD	*p* Value*
3-Hydroxy-1-methylpropylmercapuric acid (HMPMA) (µg/24 h)	Crotonaldehyde	Vuse users	98	94.68	46.606	< 0.0001	93	95.32	47.503	< 0.0001
		Smokers	40	295.85	179.032		40	295.85	179.032	
3-Hydroxypropylmercapturic acid (3-HPMA) (µg/24 h)	Acrolein	Vuse users	98	271.48	96.897	< 0.0001	93	272.24	98.859	< 0.0001
		Smokers	40	838.47	595.195		40	838.47	595.195	
3-Hydroxybenzo[a]pyrene (3-OH-B[a]P) (pg/24 h)	B[a]P	Vuse users	98	54.86	40.748	< 0.0001	93	55.17	40.138	< 0.0001
		Smokers	40	161.99	137.550		40	161.99	137.550	
Monohydroxybutenylmercapturic acid (MHBMA) (µg/24 h)	1,3-butadiene	Vuse users	98	0.37	0.164	< 0.0001	93	0.37	0.164	< 0.0001
		Smokers	40	1.56	1.167		40	1.56	1.167	
Total N-nitrosonornicotine (NNN) (ng/24 h)	NNN	Vuse users	98	0.56	0.490	< 0.0001	93	0.58	0.497	< 0.0001
		Smokers	40	2.56	2.249		40	2.56	2.249	
S-phenylmercapturic acid (S-PMA) (µg/24 h)	Benzene	Vuse users	98	0.20	0.201	< 0.0001	93	0.18	0.126	< 0.0001
		Smokers	40	3.34	2.523		40	3.34	2.523	
Total nicotine equivalents (TNeq) (mg/24 h)	Nicotine	Vuse users	98	6.48	6.154	0.0059	93	6.54	6.304	0.0074
		Smokers	40	9.70	7.216		40	9.70	7.216	
Total 4-(methylnitrosamino)-1-(3-pyridyl)-1-butanol (NNAL) (ng/24 h)	NNK	Vuse users	98	8.43	13.560	< 0.0001†	93	6.11	6.484	< 0.0001†
		Smokers	40	109.47	93.455		40	109.47	93.455	

*B[a]P* Benzo[a]pyrene, *NNK* Nicotine-derived nitrosamine ketone, *N* Number of subjects, *SD* Standard deviation

^*^Unless indicated, secondary endpoint with threshold of statistical significance = 0.05

^†^Primary endpoint with threshold of statistical significance = 0.00714, to account for multiple endpoint testing

### Biomarkers of potential harm

**Fig. 2:**
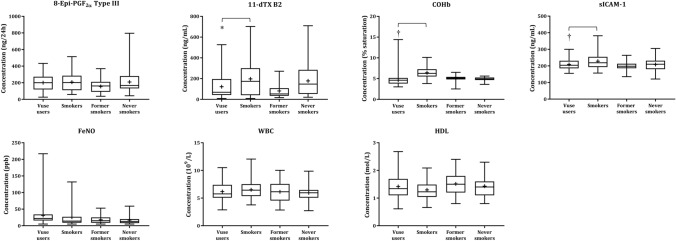
Biomarkers of potential harm boxplots by study groups for the CEVal-compliant population. The bar inside the box is the median and the arithmetic mean is the cross inside the box. The upper (75th percentile) and lower (25th percentile) sides of the box represent the interquartile range; the lower whisker is the minimum and the upper whisker is the maximum. *Secondary endpoint with threshold of statistical significance = 0.05. †Primary endpoint with threshold of statistical significance = 0.00714, to account for multiple endpoint testing

The levels of the seven BoPH assessed in the CEVal-compliant participants are shown in Fig. 2. The statistical analyses of the BoPH were performed on the Vuse user and smoker groups, as per the SAP, and the descriptive statistics and statistical analyses of the differences between these groups in the per-protocol and CEVal-compliant populations are presented in Table 2. The levels of 11-dTX B2 were significantly lower (*p* = 0.0012) in the CEVal-compliant Vuse users group when compared with people who smoke (mean ± SD; 121.95 ± 106.709 vs 196.72 ± 169.241 ng/24h, respectively). In addition, both COHb and sICAM-1 levels were observed to be significantly lower (*p* < 0.0001 and *p* = 0.0028, respectively) in the CEVal-compliant Vuse users when compared with people who smoke (mean ± SD; 4.62 ± 1.319 vs 6.42 ± 1.456 % saturation and 208.78 ± 31.665 vs 228.40 ± 44.986 ng/mL, respectively). Analysis of the WBC and 8-epi-PGF_2_ Type III determined that levels in the CEVal-compliant Vuse users group were lower than that of people who smoke (mean ± SD; 6.19 ± 1.629 vs 6.51 ±1.542 x10^9^/L and 200.10 ± 94.095 vs 206.88 ± 110.541 ng/24h, respectively); however, the difference was not statistically significant. Favourable differences in the levels of FeNO and HDL were also observed in the CEVal-compliant Vuse users compared to people who smoke (mean ± SD; 31.05 ± 31.255 vs 25.78 ± 31.764 ppb and 1.42 ± 0.405 vs 1.30 ±0.329 mmol/L, respectively); however, the differences were not statistically significant. The per-protocol population BoPH levels are presented in Supplementary Fig 3. Descriptive statistics of BoPH levels in the former smoker and never smoker groups (both per-protocol and CEVal-compliant) are presented in Supplementary Table 3.

**Fig. 3:**
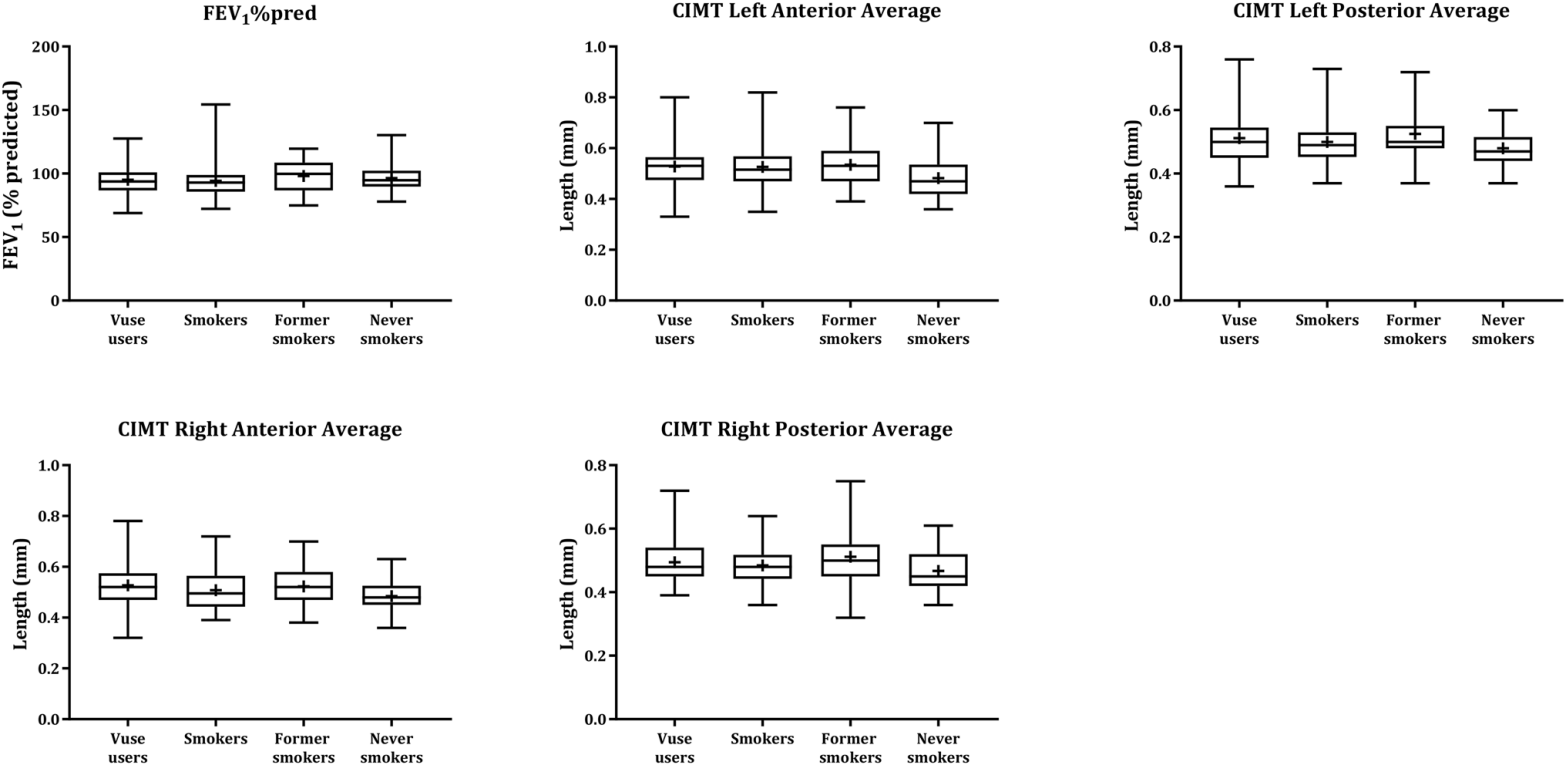
Physiological measurement boxplots by study groups for the CEVal-compliant population. The bar inside the box is the median and the arithmetic mean is the cross inside the box. The upper (75th percentile) and lower (25th percentile) sides of the box represent the inter quartile range; the lower whisker is the minimum and the upper whisker is the maximum

**Table 2:** Biomarkers of potential harm descriptive statistics and statistical analysis of the per-protocol and CEVal-compliant populations

Biomarker of potential harm (units)	Associated		Per-protocol population				CEVal-compliant population			
	Biological process	Group	*N*	Mean	SD	*p* value*	*N*	Mean	SD	*p* value*
8-Epi-prostaglandin F_2_ Type III (8-epi-PGF_2α_ Type III) (ng/24 h)	Oxidative stress	Vuse users	98	197.85	92.238	0.4604	93	200.10	94.095	0.5117
		Smokers	40	206.88	110.541		40	206.88	110.541	
11-Dehydrothromboxane B2 (11-dTX B2) (ng/24 h)	CVD	Vuse users	98	124.03	106.141	0.0014†	93	121.95	106.709	0.0012†
		Smokers	40	196.72	169.241		40	196.72	169.241	
Carboxyhaemoglobin (COHb) (% saturation)	CVD	Vuse users	98	4.65	1.313	< 0.0001	93	4.62	1.319	< 0.0001
		Smokers	40	6.42	1.456		40	6.42	1.456	
Soluble intercellular adhesion molecule-1 (sICAM-1) (ng/mL)	CVD	Vuse users	98	210.08	32.228	0.0057	93	208.78	31.665	0.0028
		Smokers	40	228.40	44.986		40	228.40	44.986	
Fractional exhaled nitric oxide (FeNO) (ppb)	COPD	Vuse users	98	30.56	30.671	0.4716	93	31.05	31.255	0.4359
		Smokers	40	25.78	31.764		40	25.78	31.764	
White blood cell count (WBC) (× 10^9^/L)	Inflammation	Vuse users	97	6.21	1.594	0.3829	92	6.19	1.629	0.3664
		Smokers	40	6.51	1.542		40	6.51	1.542	
High-density lipoprotein (HDL) cholesterol ([mmol/L)	CVD	Vuse users	98	1.41	0.401	0.0396	93	1.42	0.405	0.0256
		Smokers	40	1.30	0.329		40	1.30	0.329	

*N* number of subjects, *SD* standard deviation

^*^Unless indicated the threshold of statistical significance is 0.00714 to account for multiple endpoint testing in the primary endpoints

^†^Secondary endpoint with threshold of statistical significance = 0.05

### Physiological measurements

The results of the physiological measurements FEV1%pred and CIMT for the Vuse user and smoker groups for the CEVal-compliant population are presented in Fig. 3. The statistical analyses of the physiological measurements were performed on the Vuse user and smoker groups as per the SAP, and the descriptive statistics and statistical analyses of the differences between these groups in the per-protocol and CEVal-compliant populations are presented in Table 3. The FEV1%pred levels and CIMT values were similar for the Vuse user and smoker groups, and no statistical differences were observed for either the per-protocol or CEVal-compliant populations. The per-protocol population results for the physiological measurements are presented in Supplementary Fig 4.  Descriptive statistics of the physiological measurement results for the former smoker and never smoker groups (both per-protocol and CEVal-compliant) are presented in Supplementary Table 3.

**Table 3:** Physiological measurements’ descriptive statistics and statistical analysis of the per-protocol and CEVal-compliant populations

Physiological measurements (units)	Associated		Per-protocol population				CEVal-compliant population			
	Biological process	Group	*N*	Mean	SD	*p* value*	*N*	Mean	SD	*p* value*
Forced expiratory volume in 1 s as % of predicted (FEV1%pred) (%)	COPD	Vuse users	98	94.92	11.455	0.8032	93	95.07	11.472	0.7457
		Smokers	40	94.17	13.349		40	94.17	13.349	
Carotid intima-media thickness (CIMT) Left anterior average (mm)	CVD	Vuse users	98	0.525	0.0804	0.8902	93	0.528	0.0807	0.9829
		Smokers	40	0.527	0.0964		40	0.527	0.0964	
CIMT left posterior average (mm)	CVD	Vuse users	98	0.508	0.0771	0.4863	93	0.512	0.0774	0.3894
		Smokers	40	0.500	0.0718		40	0.500	0.0718	
CIMT right anterior average (mm)	CVD	Vuse users	98	0.523	0.0831	0.3418	93	0.527	0.0833	0.2624
		Smokers	40	0.508	0.0810		40	0.508	0.0810	
CIMT right posterior average (mm)	CVD	Vuse users	98	0.492	0.0682	0.5151	93	0.495	0.0683	0.3939
		Smokers	40	0.484	0.0569		40	0.484	0.0569	

*N* number of subjects, *SD* standard deviation

^*^Secondary endpoint with threshold of statistical significance = 0.05

### Quality of life

The statistical analysis of the Quality-of-Life questionnaire was performed on the General Health score of the Vuse user and smoker groups, as per the SAP, and the descriptive statistics and statistical analyses of the differences between these groups in the per-protocol and CEVal-compliant populations are presented in Supplementary Table 4. While General Health scores were higher in the Vuse users compared to people who smoke for both the per-protocol and CEVal-compliant populations, the difference between the Vuse users and people who smoke was not statistically significant for the per-protocol population (*p* = 0.0548). However, the higher score for the Vuse users was statistically significant for the CEVal-compliant population (*p* = 0.0425) when compared with people who smoke. Descriptive statistics of the Quality-of-Life questionnaire results for the former smoker and never smoker groups (both per-protocol and CEVal-compliant) are presented in Supplementary Table 5.

### Adverse events

There were two AEs reported during the study. Both AEs presented as headaches, were determined to be mild in severity, and were coded with the preferred terms hangover and headache.

## Discussion

The aerosol of ECs contains substantially fewer and greatly reduced levels of HPHCs as compared to cigarettes [16, 18], indicating that these nicotine products may have a role to play in a THR approach [19, 22]. This cross-sectional study aimed to add to the current knowledge regarding the role of ECs in THR, by assessing BoE and BoPH levels in individuals who had been exclusively using Vuse ECs (for at least 6 months), individuals who smoke on a daily basis, former, and never smokers. The results of this cross-sectional study found significantly lower levels of all urinary BoE; HMPMA, 3-HPMA, 3-OH-B[a]P, MHBMA, total NNN, S-PMA, total NNAL (all *p* < 0.0001), and TNeq (p = 0.0074) in CEVal-compliant participants who exclusively use Vuse EC when compared with participants who smoke on a daily basis. Additionally, urinary NNAL is associated with lung cancer risk [46], and, therefore, is considered a BoPH for lung cancer [47, 48]. Furthermore, in CEVal-compliant participants who exclusively use Vuse EC, there were significantly lower levels of the BoPH, COHb (p < 0.0001), sICAM-1 (p = 0.0028), and 11-dTX B2 (p = 0.0012), when compared with participants who smoke on a daily basis.

Several studies have evaluated the levels of BoE to HPHCs in individuals who use EC. Five short-term confinement studies [27–31] have measured BoE changes in individuals who smoke switching exclusively to ECs and those continuing to smoke. These studies all observed substantial reductions in BoE (up to 97%), including NNN, NNAL, 3-HPMA, MHBMA, S-PMA, HMPMA, CEMA, 1-OHP, and COHb, for participants switching to exclusive EC use over the study periods (5–9 days), with no significant change from baseline for those who continued to smoke. A community-based switching study also found significant reductions in eight BoE for smokers who switched exclusively to ECs for 8 weeks [51].

To date, two cross-sectional studies looking at longer term EC use (≥ 6 months) have been conducted. Shahab et al. reported that individuals who use EC had significantly lower levels of NNAL and BoE for volatile organic compounds (including metabolites of the toxins acrolein; acrylamide; acrylonitrile; 1,3-butadiene; and ethylene oxide) than solus combustible cigarette smokers [52]. However, the author noted the limitations of this study included the small sample size, which was unable to allow more sophisticated data analysis, and the limited number of biomarkers assessed. These data have also been used in a secondary analysis by Smith et al., showing that individuals who use EC exclusively had lower levels of toxicant biomarkers, but higher levels of nicotine biomarkers than individuals who smoke [33]. The second cross-sectional study [32] reported four BoE (total NNAL, 3-HPMA, COHb, and TNeq) were 46% to 86% lower in individuals who use EC vs individuals who smoke. This is the only other study to date that has measured BoPH in EC users; of the five BoPH measured, three were lower in individuals who use EC exclusively than in individuals who smoke: 11-dTX B2 (29% lower), 8-epi-PGF2_α_ Type III (23% lower), and sICAM-1 (16% lower); with no significant difference in WBC or HDL. Recent publications by Wilson et al. attempted to estimate the potential relative harm to health from using modern ECs use compared with smoking [53]. Using data from five studies comparing solus EC use with smoking, they attempted to estimate the relative disease harm of EC use compared with smoking. Due to several limitations, the study concluded that it is premature to develop quantitative estimates of the relative harm to health from using EC compared to tobacco smoking [53, 54]. However, an independent evidence review in 2018 by Public Health England concluded that EC use is around 95% less harmful than smoking [55], and recently in their 2022 report the UK Office for Health Improvement and Disparities (formerly Public Health England) “that vaping poses only a small fraction of the risks of smoking” [56], though the validity of the original estimate has been challenged citing the lack of evidence available at time the estimate was made [57].

The cross-sectional study reported here provides a comprehensive analysis of 17 BoE to HPHCs, BoPH, and physiological measures associated with biological processes linked to the potential development of oxidative stress, cardiovascular and respiratory diseases, and cancer in individuals who use EC exclusively, individuals who smoke, former smokers, and never smokers. Moreover, in contrast to many of the above studies, which relied solely on participants self-identifying as EC users or smokers, the present study has the strength that participants who self-reported as exclusive EC users or former smokers had their compliance assessed using a relatively long-term biomarker of compliance, CEVal. In addition, this study assessed BoE and BoPH in individuals who use EC exclusively that had self-selected, rather than in individuals who smoke and have been asked to switch to a specific investigational product for a short period of time. Finally, the total number of participants recruited to the study (*n* = 213) is substantially higher than that in many of the previous studies.

We note that the present study has some limitations to the study design. The cross-sectional study design provides an assessment of the study population at a specific time. In contrast, longitudinal studies repeatedly assess the same subjects to determine potential changes that occur over the study period. Typically, longitudinal studies investigating novel tobacco and nicotine products involve switching a population of smokers from combustible cigarettes to novel products and assessing changes in BoE and BoPH in subjects over time. A longitudinal design allows the baseline data of compliant subjects, switching to a novel tobacco or nicotine product, to act as their own control. In contrast, cross-sectional studies compare separate populations and those populations may have different lifestyles and behaviours that could have an effect on BoE and/or BoPH.

Collectively, the data show, for the BoE measured, that individuals who use EC exclusively were exposed to lower levels of the tobacco smoke toxicants when compared with individuals who smoke. Statistically significantly lower levels of three of the BoPH measured (COHb, sICAM-1, and 11-dTX B2) were observed in individuals who use EC exclusively compared with individuals who smoke. Though not statistically significant, directionally favourable differences were also observed for FeNO, WBC, HDL, and 8-epi PGF_2α_ Type III. The results of this study add to the current knowledge and support the role of ECs in THR.

## Supplementary Information

Below is the link to the electronic supplementary material.

Supplementary file1 (DOCX 166 kb)

## Data Availability

BAT is committed to the responsible sharing of data with the wider research community. Data access is administered for this study through an internal Data Sharing Committee on reasonable request following completion of a data sharing request form and, if applicable, a Data Access Agreement. Requests for data sharing in the first instance should be emailed to the corresponding author.
