# Supplementary material for: Biomarkers of exposure and potential harm in exclusive users of electronic cigarettes and current, former, and never smokers
Source: Intern Emerg Med. 2023 May 30;18(5):1359–71. doi: 10.1007/s11739-023-03294-9 (PMC10412681; doi:10.1007/s11739-023-03294-9)
Supplement: Supplementary file 1 — Supplementary file1 (DOCX 166 kb) [file 11739_2023_3294_MOESM1_ESM.docx]

**Supplementary Information** Tobacco & Nicotine Use History Questionnaires

**Tobacco & Nicotine Use History Questionnaire For Potential Vuse Users**

1. Are you a vaper/e-cigarette user?

☐ Yes

☐ No

1. Apart from vaping devices/e-cigarettes, have you used any other form of nicotine or tobacco product in the previous 6 months?

☐ Yes

☐ No

1. What brand and type of e-cigarette/vaping device do you use most?

Brand: ______________________

Type: ______________________

1. How often do you use this brand and type of vaping device?

☐ Daily

☐ Less than daily

1. For approximately how long have you been using this brand and type of e-cigarette/vaping device?

_____ years and _____ months

1. Are you a ‘non-inhaler’, i.e. do you usually draw vapour/aerosol from e-cigarettes/vaping devices into your mouth and throat but do not inhale?

☐ Yes

☐ No

1. Which flavour pods, cartridges or e-juice do you most often use?

______________________

1. Which level/strength of nicotine do you most often use?

**Tobacco & Nicotine Use History Questionnaire For Potential Smokers**

1. Do you smoke cigarettes?

☐ Yes

☐ No

1. Are you a ‘non-inhaler’, i.e. do you usually draw smoke from cigarettes into your mouth and throat but do not inhale?

☐ Yes

☐ No

1. For approximately how many years have you smoked cigarettes?

_____ years and _____ months

1. On average, how many cigarettes do you smoke per day?

_____ cigarettes per day

1. What is your usual brand and variant of cigarettes?

Brand: ______________________

Variant: ______________________

**Tobacco & Nicotine Use History Questionnaire For Potential Former Smokers**

1. Did you previously smoke cigarettes?

☐ Yes

☐ No

1. Have you completely quit smoking?

☐ Yes

☐ No

1. Have you used any form of nicotine or tobacco product in the previous 6 months?

☐ Yes

☐ No

1. Approximately how long ago did you completely quit smoking cigarettes?

_____ years and _____ months

**Tobacco & Nicotine Use History Questionnaire For Potential Never smokers**

1. Have you ever smoked cigarettes?

☐ Yes

☐ No

1. If yes, approximately how many cigarettes have you smoked in your whole lifetime?

_____ cigarettes

1. Have you used any form of nicotine or tobacco product in the previous 6 months?

☐ Yes

☐ No

**Supplementary Fig 1** Flow diagram of subject disposition

**Supplementary Table 1** Study participant demographic data for the CEVal-compliant population

|  |  | Vuse users | Smokers | Former smokers | Never smokers | Overall | |
| --- | --- | --- | --- | --- | --- | --- | --- |
|  |  | N=93 | N=40 | N=35 | N=37 | N=205 | |
| Sex |  |  |  |  |  |  | |
| Female | n (%) | 33 (35.5) | 17 (42.5) | 18 (51.4) | 17 (45.9) | 85 (41.5) | |
| Male | n (%) | 60 (64.5) | 23 (57.5) | 17 (48.6) | 20 (54.1) | 120 (58.5) | |
| Age (years) | Mean (SD) | 29.6 (8.26) | 29.5 (6.57) | 35.8 (9.73) | 30.4 (7.64) | 30.8 (8.40) | |
| Weight (kg) | Mean (SD) | 74.24 (12.933) | 73.25 (11.830) | 71.95 (9.868) | 68.89 (10.695) | 72.69 (11.936) | |
| Body mass index (kg/m^2^) | Mean (SD) | 24.32 (3.093) | 24.38 (2.813) | 24.07 (2.838) | 23.04 (2.496) | 24.06 (2.917) | |
| Race |  |  |  |  |  |  | |
| Asian | n (%) | 21 (22.6) | 3 (7.5) | 2 (5.7) | 6 (16.2) | 32 (15.6) | |
| Black/African American | n (%) | 10 (10.8) | 4 (10.0) | 2 (5.7) | 5 (13.5) | 21 (10.2) | |
| Caucasian | n (%) | 18 (19.4) | 7 (17.5) | 7 (20.0) | 14 (37.8) | 46 (22.4) | |
| Other | n (%) | 10 (10.8) | 7 (17.5) | 5 (14.3) | 2 (5.4) | 24 (11.7) | |
| White | n (%) | 34 (36.6) | 19 (47.5) | 19 (54.3) | 10 (27.0) | 82 (40.0) | |
| Ethnicity |  |  |  |  |  |  | |
| Hispanic/Latino | n (%) | 8 (8.6) | 1 (2.5) | 1 (2.9) | 0 | 10 (4.9) | |
| Not Hispanic/Latino | n (%) | 85 (91.4) | 39 (97.5) | 34(97.1) | 37 (100.0) | 195 (95.1) | |
| How long using e-cigarette/vaping device? (years) | Mean (SD) | 1.14 (0.721) | N/A | N/A | N/A | N/A | |
| Number of years smoking | Mean (SD) | N/A | 11.68 (6.929) | N/A | N/A | N/A | |
| Number of cigarettes per day | Mean (SD) | N/A | 12.25 (2.519) | N/A | N/A | N/A | |
| How long ago completely quit smoking cigarettes? (years) | Mean (SD) | N/A | N/A | 4.62 (5.200) | N/A | N/A | |
| N – Number of subjects, SD – Standard deviation | | | | | | |  |

**Supplementary Table 2** Study participant demographic data for the per-protocol population

|  |  | | Vuse users | | | Smokers | Former smokers | Never smokers | | Overall | |
| --- | --- | --- | --- | --- | --- | --- | --- | --- | --- | --- | --- |
|  |  | | N=98 | | | N=40 | N=37 | N=37 | | N=212 | |
| Sex |  | |  | | |  |  |  | |  | |
| Female | n (%) | | 36 (36.7) | | | 17 (42.5) | 19 (51.4) | 17 (45.9) | | 89 (42.0) | |
| Male | n (%) | | 62 (63.3) | | | 23 (57.5) | 18 (48.6) | 20 (54.1) | | 123 (58.0) | |
| Age (years) | Mean (SD) | | 29.4 (8.22) | | | 29.5 (6.57) | 35.2 (9.80) | 30.4 (7.64) | | 30.6 (8.37) | |
| Weight (kg) | Mean (SD) | | 73.72 (13.033) | | | 73.25 (11.830) | 71.83 (9.879) | 68.89 (10.695) | | 72.46 (11.970) | |
| Body mass index (kg/m^2^) | Mean (SD) | | 24.27 (3.153) | | | 24.38 (2.813) | 24.10 (2.762) | 23.04 (2.496) | | 24.05 (2.936) | |
| Race |  | |  | | |  |  |  | |  | |
| Asian | n (%) | | 22 (22.4) | | | 3 (7.5) | 2 (5.4) | 6 (16.2) | | 33 (15.6) | |
| Black/African American | n (%) | | 11 (11.2) | | | 4 (10.0) | 2 (5.4) | 5 (13.5) | | 22 (10.4) | |
| Caucasian | n (%) | | 20 (20.4) | | | 7 (17.5) | 7 (18.9) | 14 (37.8) | | 48 (22.6) | |
| Other | n (%) | | 10 (10.2) | | | 7 (17.5) | 6 (16.2) | 2 (5.4) | | 25 (11.8) | |
| White | n (%) | | 35 (35.7) | | | 19 (47.5) | 20 (54.1) | 10 (27.0) | | 84 (39.6) | |
| Ethnicity |  | |  | | |  |  |  | |  | |
| Hispanic/Latino | n (%) | | 8 (8.2) | | | 1 (2.5) | 3 (8.1) | 0 | | 12 (5.7) | |
| Not Hispanic/Latino | n (%) | | 90 (91.8) | | | 39 (97.5) | 34 (91.9) | 37 (100.0) | | 200 (94.3) | |
| How long using e-cigarette/vaping device? (years) | Mean (SD) | | 1.13 (0.713) | | | N/A | N/A | N/A | | N/A | |
| Number of years smoking | Mean (SD) | | N/A | | | 11.68 (6.929) | N/A | N/A | | N/A | |
| Number of cigarettes per day | Mean (SD) | | N/A | | | 12.25 (2.519) | N/A | N/A | | N/A | |
| How long ago completely quit smoking cigarettes? (years) | Mean (SD) | | N/A | | | N/A | 4.41 (5.139) | N/A | | N/A | |
| N – Number of subjects, SD – Standard deviation | |  | |  |  | |  | |  | |  |

**Supplementary Table 3** Biomarker descriptive statistics of the per-protocol and CEVal-compliant populations

| Biomarker of exposure |  | Per-protocol population | | | CEVal-compliant population | | | |
| --- | --- | --- | --- | --- | --- | --- | --- | --- |
| [units] | Group | N | Mean | SD | N | Mean | SD | |
| 3-hydroxy-1-methylpropylmercapuric acid (HMPMA) | Vuse users | 98 | 94.68 | 46.606 | 93 | 95.32 | 47.503 | |
| [µg/24h] | Smokers | 40 | 295.85 | 179.032 | 40 | 295.85 | 179.032 | |
|  | Former smokers | 37 | 84.81 | 28.647 | 35 | 86.24 | 28.769 | |
|  | Never smokers | 37 | 97.32 | 26.872 | 37 | 97.32 | 26.872 | |
| 3-hydroxypropylmercapturic acid (3-HPMA) | Vuse users | 98 | 271.48 | 96.897 | 93 | 272.24 | 98.859 | |
| [µg/24h] | Smokers | 40 | 838.47 | 595.195 | 40 | 838.47 | 595.195 | |
|  | Former smokers | 37 | 270.75 | 126.325 | 35 | 274.71 | 128.814 | |
|  | Never smokers | 37 | 311.68 | 209.009 | 37 | 311.68 | 209.009 | |
| 3-hydroxybenzo[a]pyrene (3-OH-B[a]P) | Vuse users | 98 | 54.86 | 40.748 | 93 | 55.17 | 40.138 | |
| [pg/24h] | Smokers | 40 | 161.99 | 137.550 | 40 | 161.99 | 137.550 | |
|  | Former smokers | 37 | 59.00 | 58.985 | 35 | 60.82 | 60.168 | |
|  | Never smokers | 37 | 135.78 | 433.211 | 37 | 135.78 | 433.211 | |
| Monohydroxybutenylmercapturic acid (MHBMA) | Vuse users | 98 | 0.37 | 0.164 | 93 | 0.37 | 0.164 | |
| [µg/24h] | Smokers | 40 | 1.56 | 1.167 | 40 | 1.56 | 1.167 | |
|  | Former smokers | 37 | 0.35 | 0.174 | 35 | 0.35 | 0.175 | |
|  | Never smokers | 37 | 0.35 | 0.218 | 37 | 0.35 | 0.218 | |
| Total N-nitrosonornicotine (NNN) | Vuse users | 98 | 0.56 | 0.490 | 93 | 0.58 | 0.497 | |
| [ng/24h] | Smokers | 40 | 2.56 | 2.249 | 40 | 2.56 | 2.249 | |
|  | Former smokers | 37 | 0.62 | 0.774 | 35 | 0.64 | 0.789 | |
|  | Never smokers | 37 | 0.51 | 0.309 | 37 | 0.51 | 0.309 | |
| S-phenylmercapturic acid (S-PMA) | Vuse users | 98 | 0.20 | 0.201 | 93 | 0.18 | 0.126 | |
| [µg/24h] | Smokers | 40 | 3.34 | 2.523 | 40 | 3.34 | 2.523 | |
|  | Former smokers | 37 | 0.13 | 0.050 | 35 | 0.13 | 0.051 | |
|  | Never smokers | 37 | 0.14 | 0.049 | 37 | 0.14 | 0.049 | |
| Total nicotine equivalents (TNeq) | Vuse users | 98 | 6.48 | 6.154 | 93 | 6.54 | 6.304 | |
| [mg/24h] | Smokers | 40 | 9.70 | 7.216 | 40 | 9.70 | 7.216 | |
|  | Former smokers | 37 | 0.02 | 0.010 | 35 | 0.02 | 0.009 | |
|  | Never smokers | 37 | 0.02 | 0.012 | 37 | 0.02 | 0.012 | |
| Total 4-(methylnitrosamino)-1-(3-pyridyl)-1-butanol (NNAL) | Vuse users | 98 | 8.43 | 13.560 | 93 | 6.11 | 6.484 | |
| [ng/24h] | Smokers | 40 | 109.47 | 93.455 | 40 | 109.47 | 93.455 | |
|  | Former smokers | 37 | 2.74 | 1.982 | 35 | 2.36 | 1.160 | |
|  | Never smokers | 37 | 2.63 | 1.890 | 37 | 2.63 | 1.890 | |
| Biomarker of potential harm |  | Per-protocol population | | | CEVal-compliant population | | | |
| [units] | Group | N | Mean | SD | N | Mean | SD | |
| 8-epi-prostaglandin F_2_ Type III (8-epi-PGF_2α_ Type III) | Vuse users | 98 | 197.85 | 92.238 | 93 | 200.10 | 94.095 | |
| [ng/24h] | Smokers | 40 | 206.88 | 110.541 | 40 | 206.88 | 110.541 | |
|  | Former smokers | 37 | 154.40 | 81.308 | 35 | 155.54 | 82.938 | |
|  | Never smokers | 37 | 208.97 | 142.431 | 37 | 208.97 | 142.431 | |
| 11-dehydrothromboxane B2 (11-dTX B2) | Vuse users | 98 | 124.03 | 106.141 | 93 | 121.95 | 106.709 | |
| [ng/24h] | Smokers | 40 | 196.72 | 169.241 | 40 | 196.72 | 169.241 | |
|  | Former smokers | 37 | 81.72 | 71.228 | 35 | 80.89 | 71.037 | |
|  | Never smokers | 37 | 177.48 | 153.646 | 37 | 177.48 | 153.646 | |
| Carboxyhaemoglobin (COHb) | Vuse users | 98 | 4.65 | 1.313 | 93 | 4.62 | 1.319 | |
| [% saturation] | Smokers | 40 | 6.42 | 1.456 | 40 | 6.42 | 1.456 | |
|  | Former smokers | 37 | 5.11 | 0.678 | 35 | 5.12 | 0.682 | |
|  | Never smokers | 37 | 4.84 | 0.536 | 37 | 4.84 | 0.536 | |
| Soluble intercellular adhesion molecule-1 (sICAM-1) | Vuse users | 98 | 210.08 | 32.228 | 93 | 208.78 | 31.665 | |
| [ng/mL] | Smokers | 40 | 228.40 | 44.986 | 40 | 228.40 | 44.986 | |
|  | Former smokers | 37 | 196.76 | 27.622 | 35 | 198.40 | 26.712 | |
|  | Never smokers | 37 | 208.59 | 36.700 | 37 | 208.59 | 36.700 | |
| Fractional exhaled nitric oxide (FeNO) | Vuse users | 98 | 30.56 | 30.671 | 93 | 31.05 | 31.255 | |
| [ppb] | Smokers | 40 | 25.78 | 31.764 | 40 | 25.78 | 31.764 | |
|  | Former smokers | 37 | 17.30 | 11.372 | 35 | 17.30 | 11.372 | |
|  | Never smokers | 37 | 16.89 | 12.821 | 37 | 16.89 | 12.821 | |
| White blood cell count (WBC) | Vuse users | 97 | 6.21 | 1.594 | 92 | 6.19 | 1.629 | |
| [x10^9^/L] | Smokers | 40 | 6.51 | 1.542 | 40 | 6.51 | 1.542 | |
|  | Former smokers | 37 | 6.12 | 1.689 | 35 | 6.13 | 1.702 | |
|  | Never smokers | 37 | 5.95 | 1.470 | 37 | 5.95 | 1.470 | |
| High-density lipoprotein (HDL) cholesterol | Vuse users | 98 | 1.41 | 0.401 | 93 | 1.42 | 0.405 | |
| [mmol/L] | Smokers | 40 | 1.30 | 0.329 | 40 | 1.30 | 0.329 | |
|  | Former smokers | 37 | 1.55 | 0.411 | 35 | 1.51 | 0.393 | |
|  | Never smokers | 37 | 1.43 | 0.406 | 37 | 1.43 | 0.406 | |
| Physiological measure |  | Per-protocol population | | | CEVal-compliant population | | | |
| [units] | Group | N | Mean | SD | N | Mean | SD | |
| Forced Expiratory Volume in 1 second as % of predicted | Vuse users | 98 | 94.92 | 11.455 | 93 | 95.07 | 11.472 | |
| (FEV1%pred) | Smokers | 40 | 94.17 | 13.349 | 40 | 94.17 | 13.349 | |
| [%] | Former smokers | 37 | 98.78 | 12.828 | 35 | 98.07 | 12.490 | |
|  | Never smokers | 37 | 96.44 | 10.445 | 37 | 96.44 | 10.445 | |
| Carotid intima-media thickness (CIMT) Left Anterior Average | Vuse users | 98 | 0.525 | 0.0804 | 93 | 0.528 | 0.0807 | |
| [mm] | Smokers | 40 | 0.527 | 0.0964 | 40 | 0.527 | 0.0964 | |
|  | Former smokers | 37 | 0.534 | 0.0821 | 35 | 0.535 | 0.0844 | |
|  | Never smokers | 37 | 0.482 | 0.0774 | 37 | 0.482 | 0.0774 | |
| CIMT Left Posterior Average | Vuse users | 98 | 0.508 | 0.0771 | 93 | 0.512 | 0.0774 | |
| [mm] | Smokers | 40 | 0.500 | 0.0718 | 40 | 0.500 | 0.0718 | |
|  | Former smokers | 37 | 0.522 | 0.0799 | 35 | 0.525 | 0.0803 | |
|  | Never smokers | 37 | 0.479 | 0.0579 | 37 | 0.479 | 0.0579 | |
| CIMT Right Anterior Average | Vuse users | 98 | 0.523 | 0.0831 | 93 | 0.527 | 0.0833 | |
| [mm] | Smokers | 40 | 0.508 | 0.0810 | 40 | 0.508 | 0.0810 | |
|  | Former smokers | 37 | 0.524 | 0.0803 | 35 | 0.523 | 0.0823 | |
|  | Never smokers | 37 | 0.485 | 0.0578 | 37 | 0.485 | 0.0578 | |
| CIMT Right Posterior Average | Vuse users | 98 | 0.492 | 0.0682 | 93 | 0.495 | 0.0683 | |
| [mm] | Smokers | 40 | 0.484 | 0.0569 | 40 | 0.484 | 0.0569 | |
|  | Former smokers | 37 | 0.512 | 0.0916 | 35 | 0.512 | 0.0941 | |
|  | Never smokers | 37 | 0.467 | 0.0611 | 37 | 0.467 | 0.0611 | |
| N – Number of subjects, SD – Standard deviation | | | | | | | |  |

**Supplementary Fig 2** Biomarkers of exposure boxplots by study groups for the per protocol population. The bar inside the box is the median and the arithmetic mean is the cross inside the box. The upper (75th percentile) and lower (25th percentile) sides of the box represent the inter quartile range, the lower whisker is the minimum and the upper whisker is the maximum. *Secondary endpoint with threshold of statistical significance = 0.05. †Primary endpoint with threshold of statistical significance = 0.00714, to account for multiple endpoint testing

†

*

*

*

*

*

*

*

†

†

*

**Supplementary Fig 3** Biomarkers of potential harm boxplots by study groups for the per protocol population. The bar inside the box is the median and the arithmetic mean is the cross inside the box. The upper (75th percentile) and lower (25th percentile) sides of the box represent the inter quartile range, the lower whisker is the minimum and the upper whisker is the maximum. *Secondary endpoint with threshold of statistical significance = 0.05. †Primary endpoint with threshold of statistical significance = 0.00714, to account for multiple endpoint testing

**Supplementary Fig 4** Physiological measurements boxplots by study groups for the per protocol population. The bar inside the box is the median and the arithmetic mean is the cross inside the box. The upper (75th percentile) and lower (25th percentile) sides of the box represent the inter quartile range, the lower whisker is the minimum and the upper whisker is the maximum

|  |  | Per-protocol population | | | | | CEVal-compliant population | | | | | |  |  |
| --- | --- | --- | --- | --- | --- | --- | --- | --- | --- | --- | --- | --- | --- | --- |
| Domain | Group | N | Mean | | SD | *p* value* | | N | | Mean | SD | *p* value* |  |  |
| General health | Vuse users | 98 | 79.23 | | 13.265 | 0.0548 | | 93 | | 79.46 | 12.757 | 0.0425 |  |  |
|  | Smokers | 40 | 74.00 | | 15.616 |  | | 40 | | 74.00 | 15.616 |  |  |  |
| Emotional well-being | Vuse users | 98 | 80.69 | | 13.665 |  | | 93 | | 80.86 | 13.792 |  |  |  |
|  | Smokers | 40 | 73.20 | | 15.510 |  | | 40 | | 73.20 | 15.510 |  |  |  |
| Energy/fatigue | Vuse users | 98 | 66.17 | | 16.619 |  | | 93 | | 66.29 | 16.614 |  |  |  |
|  | Smokers | 40 | 61.63 | | 18.341 |  | | 40 | | 61.63 | 18.341 |  |  |  |
| Pain | Vuse users | 98 | 92.17 | | 12.454 |  | | 93 | | 91.85 | 12.678 |  |  |  |
|  | Smokers | 40 | 90.56 | | 14.384 |  | | 40 | | 90.56 | 14.384 |  |  |  |
| Physical functioning | Vuse users | 98 | 95.51 | | 9.511 |  | | 93 | | 95.38 | 9.703 |  |  |  |
|  | Smokers | 40 | 91.50 | | 13.502 |  | | 40 | | 91.50 | 13.502 |  |  |  |
| Role limitations due to emotional problems | Vuse users | 98 | 89.46 | | 23.738 |  | | 93 | | 89.61 | 23.559 |  |  |  |
|  | Smokers | 40 | 85.83 | | 28.130 |  | | 40 | | 85.83 | 28.130 |  |  |  |
| Role limitations due to physical health | Vuse users | 98 | 96.68 | | 12.246 |  | | 93 | | 96.51 | 12.550 |  |  |  |
|  | Smokers | 40 | 91.25 | | 21.595 |  | | 40 | | 91.25 | 21.595 |  |  |  |
| Social functioning | Vuse users | 98 | 90.31 | | 16.207 |  | | 93 | | 90.46 | 16.214 |  |  |  |
|  | Smokers | 40 | 88.75 | | 17.172 |  | | 40 | | 88.75 | 17.172 |  |  |  |
| * Secondary endpoint with threshold of statistical significance = 0.05  N – Number of subjects, SD – Standard deviation | | | |  | | | | |  | | | | |  |

**Supplementary Table 4** Quality of life questionnaire scores descriptive statistics and statistical analysis of the per-protocol and CEVal-compliant populations

|  | |  | Per-protocol population | | | | | CEVal-compliant population | | | |
| --- | --- | --- | --- | --- | --- | --- | --- | --- | --- | --- | --- |
| Domain | Group | | | N | Mean | SD | N | | Mean | SD |  |
| General health | Former smokers | | | 37 | 83.11 | 12.547 | 35 | | 83.86 | 12.490 |  |
|  | Never smokers | | | 37 | 84.86 | 8.857 | 37 | | 84.86 | 8.857 |  |
| Emotional well-being | Former smokers | | | 37 | 80.76 | 12.359 | 35 | | 80.80 | 12.716 |  |
|  | Never smokers | | | 37 | 80.00 | 11.470 | 37 | | 80.00 | 11.470 |  |
| Energy/fatigue | Former smokers | | | 37 | 67.84 | 17.894 | 35 | | 68.14 | 18.355 |  |
|  | Never smokers | | | 37 | 72.57 | 14.124 | 37 | | 72.57 | 14.124 |  |
| Pain | Former smokers | | | 37 | 91.69 | 12.528 | 35 | | 92.43 | 11.368 |  |
|  | Never smokers | | | 37 | 95.54 | 7.220 | 37 | | 95.54 | 7.220 |  |
| Physical functioning | Former smokers | | | 37 | 95.00 | 14.044 | 35 | | 94.71 | 14.397 |  |
|  | Never smokers | | | 37 | 99.19 | 2.209 | 37 | | 99.19 | 2.209 |  |
| Role limitations due to emotional problems | Former smokers | | | 37 | 89.19 | 23.641 | 35 | | 88.57 | 24.176 |  |
|  | Never smokers | | | 37 | 90.09 | 22.034 | 37 | | 90.09 | 22.034 |  |
| Role limitations due to physical health | Former smokers | | | 37 | 94.59 | 18.759 | 35 | | 94.29 | 19.256 |  |
|  | Never smokers | | | 37 | 98.65 | 5.731 | 37 | | 98.65 | 5.731 |  |
| Social functioning | Former smokers | | | 37 | 91.55 | 14.747 | 35 | | 91.07 | 15.028 |  |
|  | Never smokers | | | 37 | 93.58 | 11.266 | 37 | | 93.58 | 11.266 |  |
| N – Number of subjects, SD – Standard deviation | | | | | | | | | | | |

**Supplementary Table 5** Quality of life questionnaire scores descriptive statistics of former smokers and never smokers from the per-protocol and CEVal-compliant populations
